# Evolutionary adaptations generally reverse phenotypic plasticity to restore ancestral phenotypes during new environment adaptation in cattle

**DOI:** 10.1002/ece3.11489

**Published:** 2024-06-04

**Authors:** Qiang Jiang, Li Zhu, Hao Zeng, Zhuzha Basang, Quji Suolang, Jinming Huang, Yafei Cai

**Affiliations:** ^1^ Department of Animal Genetics, Breeding and Reproduction, College of Animal Science and Technology Nanjing Agricultural University Nanjing China; ^2^ Institute of Animal Science and Veterinary Medicine Shandong Academy of Agricultural Sciences Jinan China; ^3^ Department of Animal Genetics, Breeding and Reproduction, College of Animal Science and Technology Yunnan Agricultural University Kunming China; ^4^ Institute of Animal Science and Veterinary Medicine Tibet Academy of Agricultural and Animal Husbandry Sciences Lhasa China

**Keywords:** cattle, environment adaptation, gene expression, phenotypic plasticity

## Abstract

Phenotype plasticity and evolution adaptations are the two main ways in which allow populations to deal with environmental changes, but the potential relationship between them remains controversial. Using a reciprocal transplant approach with cattle adapted to the Tibetan Plateau and adjacent lowlands, we aim to investigate the relative contributions of evolutionary processes and phenotypic plasticity in driving both phenotypic and transcriptomic changes under natural conditions. We observed that while numerous genetic transcriptomic changes were evident during the forward adaptation to highland environments, plastic changes predominantly facilitate the transformation of transcriptomes into a preferred state when Tibetan cattle are reintroduced to lowland habitats. Genes with ancestral plasticity are generally reversed by evolutionary adaptations and show a closer expression level to the ancestral stage in evolved Tibetan cattle. A similar trend was also observed at the phenotypes level, with a majority of biochemical and hemorheology phenotypes showing a tendency to revert to their ancestral patterns, suggesting the restoration of ancestral expression levels is a widespread evolutionary trend during adaptation. The findings of our study contribute to the debate regarding the relative contributions of plasticity and genetic changes in mammal environment adaptation. Furthermore, we highlight that the restoration of ancestral phenotypes represents a general pattern in cattle new environment adaptation.

## Abstract 2

Our findings reveal that while numerous genetic transcriptomic changes were observed during the forward adaptation to highland environments, plastic changes predominantly facilitate the transformation of transcriptomes into a preferred state when Tibetan cattle are reintroduced to lowland habitats. Genes exhibiting ancestral plasticity generally undergo reversal through evolutionary adaptations, resulting in closer expression levels resembling their ancestral stage in evolved Tibetan cattle. A similar trend was also observed at the phenotype level, where a majority of biochemical and hemorheology traits displayed a tendency to revert to their ancestral patterns, suggesting that restoration of ancestral expression levels is a widespread evolutionary trend during adaptation.

## 1 INTRODUCTION

A significant challenge in contemporary biology lies in comprehending the mechanisms by which organisms acclimate to alterations in their surroundings and the emergence of intricate, adaptive characteristics. Both phenotypic plasticity and genetic adaptation can contribute to phenotypic evolution response to different environments. Phenotypic plasticity refers to the capacity of a specific genotype to generate various phenotypes that respond to environmental factors. It is commonly witnessed as an immediate response of individuals to changes in their environment. Conversely, evolution encompasses modifications in allele frequencies among a population. This process occurs over numerous generations and represents a longer‐lasting reaction that can result in local adaptation. However, determining the proportion of observed trait differentiation attributed to environmentally induced phenotype plasticity or genetically based adaptive evolution in slowly changing environments is challenging due to the varying contributions expected from these factors depending on the spatial grain of environmental variation (Baythavong, 2011). The challenge can be surmounted through the investigation of adaptation to more sudden shifts in the environment, such as acclimation to hypoxia. In animal species inhabiting regions characterized by steep elevational gradients, they encounter escalating physiological challenges linked to hypoxia and exposure to cold temperatures as altitude increases. Furthermore, these environmental stressors can exhibit substantial variations across relatively short distances (Storz & Scott, 2019). This finely nuanced environmental diversity along elevational gradients is particularly favorable for the development of phenotypic plasticity (Storz & Scott, 2010). This is because an enhanced capacity for acclimatization enables organisms to effectively adapt to shifts in the optimal traits within their local surroundings.

Phenotype plasticity may have a dual effect during highland environment adaptation (Ghalambor et al., 2015; Ho & Zhang, 2018; Kelly, 2019). The opportunity for genetic‐based adaptation is precluded when the mean phenotype can be matched to the optimum of the new environment solely through plasticity (Ghalambor et al., 2007). Another potential explanation is that the plastic response causes a partial adjustment of the average phenotype toward the new optimal state, albeit insufficient to fully attain the phenotypic optimum, subsequent evolutionary processes then further refine the phenotype toward its optimal state (Pfennig et al., 2010). In the above two cases, plasticity is adaptive. In contrast, plasticity becomes disadvantageous when the resulting response moves the average phenotype even further from the optimal state. This creates a selective force to counteract the impact induced by environmental factors (Ho & Zhang, 2018, 2019; Koch & Guillaume, 2020; Swaegers et al., 2020).

**FIGURE 1:**
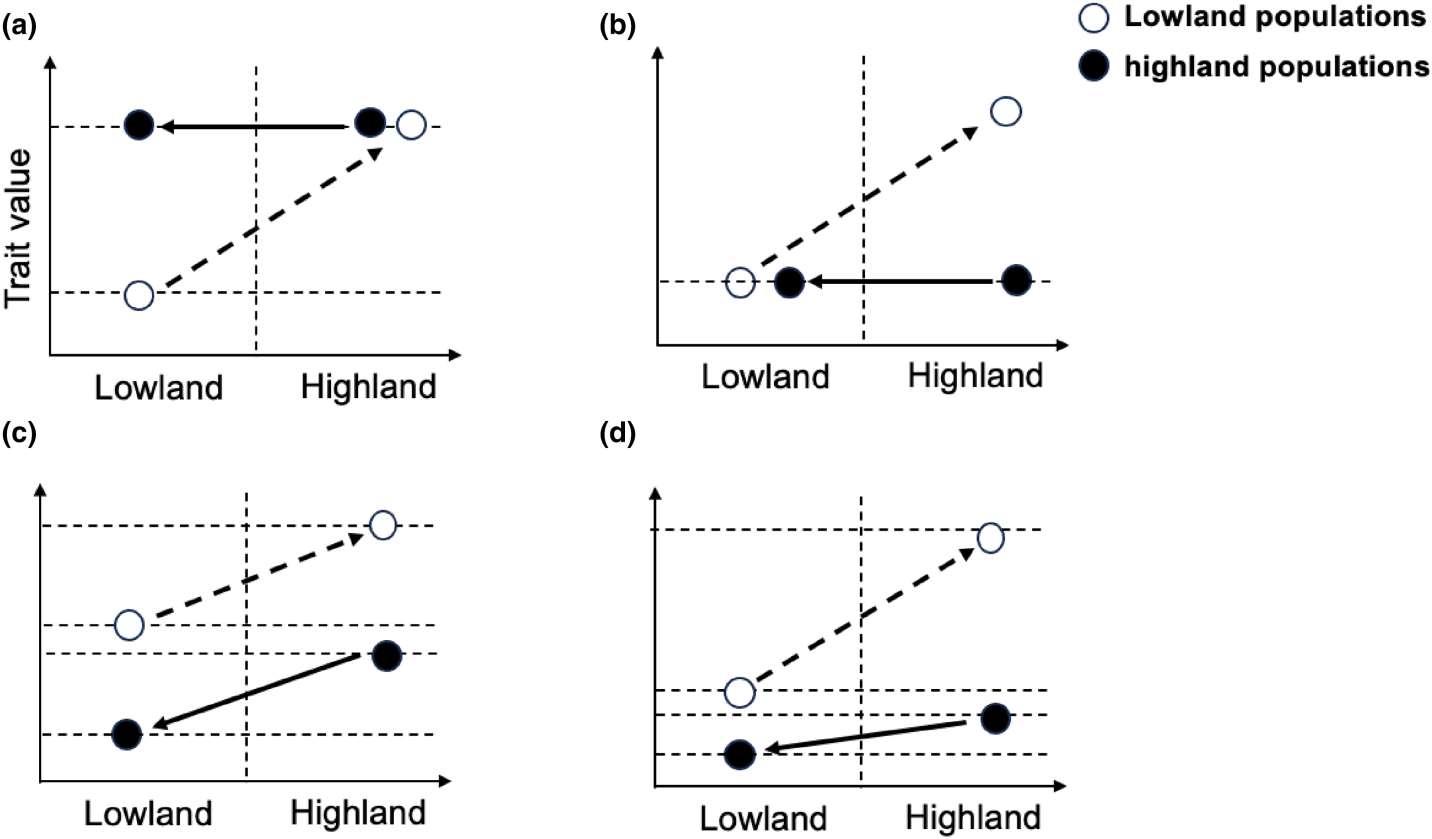
The patterns of evolutionary changes in phenotypic plasticity. (a) Genetic assimilation. The fitness function for the trait under consideration exhibits variation between the ancestral lowland habitat and the recently colonized highland environment. The plastic response induced by hypoxia is advantageous (as depicted by a solid line representing reaction norm) and subsequently becomes stabilized through selection, leading to diminished plasticity. (b) Genetic compensation with canalization. The fitness function remains consistent for the trait in both lowland and highland environments. The plastic response induced by hypoxia causes a deviation from the optimal trait value. In highland natives, selection acts on genetically determined trait variation to counterbalance the plastic alteration, thereby reinstating the ancestral phenotype (i.e., the same phenotype exhibited by lowland natives in their original environment). (c) Same scenario as in b, but the highland population evolves a new reaction to reach the optimum by changing mean expression, that is, shift in the intercept of the reaction norm without a concomitant loss of plasticity (change in Y‐intercept, but no change in slope). (d) Combination of genetic assimilation of favorable plastic responses and genetic compensation of detrimental. Highland population reached the optimum by both changing mean expression, that is, shift in the intercept and change in the plasticity, that is, the slope of the reaction norm (both change in Y‐intercept and in slope).

The interaction between plastic responses and local adaptation within a population plays a crucial role in shaping the dynamics of reaction norms during evolution (Forsman, 2015; Ghalambor et al., 2007; Wund, 2012). If a specific phenotype induced by low oxygen levels enables individuals to survive and thrive in the highland environment, maintaining consistent expression of this phenotype may provide adaptive advantages for long‐term adaptation at high altitudes. In such situations, the environmentally induced response has the potential to undergo genetic assimilation through selection, thereby becoming genetically fixed. When the genetic assimilation of adaptive plastic response occurs within a population, it results in a decrease in the level of plasticity observed in the trait that has been selected, so the reciprocal transplant result shows the pronounced difference in phenotype between high altitude population and low altitude population in their native habitats (Figure 1a). On the other hand, certain physiological traits may have one optimal value throughout the entire altitude range of a species. In such scenarios, the presence of low oxygen levels would create unfavorable variations from the overall phenotypic optimum. This would result in selection pressure that opposes the plastic response and aims to reinstate the ancestral phenotype observed at lower altitudes (Figure 1b–d).

The phenomenon where selection acts on genetically determined variation in traits to counteract an unfavorable plastic response is referred to as genetic compensation (Grether, 2005). Indeed, genetic compensation may encompass the occurrence of ancestral phenotype canalization, which refers to a modification in the inclination of the reaction norm while maintaining an unaltered Y‐intercept. In this specific scenario, the canalized phenotype is unconditionally advantageous as it confers maximum fitness in both highland and lowland environments, and the reciprocal transplant result shows no difference in the phenotype of high altitude population and low altitude population in their native habitats (Figure 1b). Another scenario is genetic compensation without canalization. The Highland population undergoes an evolutionary adaptation, wherein compensation entails an adaptive shift in the reaction norm while maintaining plasticity (change in Y‐intercept, but no alteration in slope) (Figure 1c). In such cases. However, the induced phenotype provides optimal fitness at high altitudes, its plastic response would be disadvantageous upon returning to normal oxygen conditions at low altitudes.

Therefore, genetic compensation without canalization represents an adaptive solution specifically tailored for species specialized in high‐altitude habitats rather than generalist species. Recent research findings have indicated that the physiological response to hypoxia at high altitudes entails intricate interplay among interconnected characteristics with different degrees of adaptability. This course potentially encompasses a blend of incorporating advantageous plastic responses into the genetic makeup and offsetting unfavorable responses through genetic compensation (both changes in Y‐intercept and slope) (Figure 1d).

In the present study, we performed a reciprocal transplant experiment to assess the extent to which genetic and plastic phenotypic changes contribute to cattle adaptation in natural environments. We were interested in investigating the potential synergy or antagonism between environment stress‐induced phenotypic plasticity and genetic adaptation. In this manner, we can investigate the impact of plasticity on evolution and determine if plasticity has undergone any evolutionary adaptations.

## 2 MATERIALS AND METHODS

### 2.1 Animal rearing and reciprocal transplant

We performed a reciprocal transplant approach to analyze plastic phenotype in cattle altitude adaptation. Four experimental groups were designed including two native groups and two transplant groups. Specifically, about native groups, we reared lowland cattle (Zhaotong breed, *n* = 80) in Yuanjiang (102°11′ E, 23°28′ N, altitude 300 m) and Tibetan cattle (*n* = 118) in Shangri‐la (99°85′ E, 27°78′ N, altitude 3500 m) within Yunnan Province, China. In addition, about transplant groups, we raised lowland cattle (*n* = 26) in the Shangri‐la (99°85′ E, 27°78′ N, altitude 3500 m) and Tibetan cattle (*n* = 32) in the Yuanjiang (102°11′ E, 23°28′ N, altitude 300 m). All the experimental cattle were fed on a standard pasture for 6 months to ensure that each group fully adapted to the environment. After that, we collected anticoagulant whole blood form the resting state of cattle to preform blood biochemical and hemorheology test.

### 2.2 Sequencing, assembly, and data filtering

Three oxygen‐sensitive tissues (heart, lung, and liver) were collected to perform RNA‐sequencing. The standard TRIzol method (Invitrogen) was employed for the isolation of total RNA from these tissues. RNA sequencing was performed on the HiSeq™ 4000 sequencing platform. A total of 51 libraries were built with average sequencing deep >10× using the Illumina HiSeq™ 4000 platform. Read mapping could be accurately compared to the cattle reference genome (ARS‐UCD1.2) by using HISAT2 software.

### 2.3 Gene expression plasticity

We followed the protocol published by Ho et al. (2020). First, we define the course that lowland cattle to the Tibetan Plateau as forward adaptation, and Tibetan cattle readaptation to the plain was reverse adaptation. We calculated the following expression levels of each differentially expressed gene (DEG) by using transcript per million (TPM) as expression values (Appendix S1): Lo—mean expression value of lowland cattle in lowland conditions; Lp—mean expression value of lowland cattle in highland conditions; La—mean expression value of Tibetan cattle in lowland conditions. Second, we calculated the ancestral plastic change (PCa), as the disparity in gene expression values between lowland cattle inhabiting their native lowland environment and the lowland cattle in their “foreign” highland environment. Therefore, PCa serves as the adaptive reaction of lowland cattle to the challenging highland environment. Subsequently, we computed ancestral evolutionary change (ECa) by comparing gene expression values between lowland cattle in their unfamiliar highland surroundings and highland cattle in their familiar high environment. Thus, ECa represents the subsequent evolutionary alteration in gene expression between the lowland cattle population that has adapted to a new highland environment and the ancestral highland cattle population (Figure 1c). To assess reverse adaptation, we computed the evolved plastic change (PCe) as the disparity in gene expression values between the highland cattle in its native high environment and in its non‐native lowland environment. Therefore, PCe signifies the plastic response of highland cattle to their original lowland habitat. Subsequently, we determined evolved evolutionary change (ECe) by comparing gene expression values between highland cattle residing in their non‐native lowland environment and lowland cattle living in their ancestral lowlands. Hence, ECe denotes the ensuing evolutionary modification in gene expression between the adapted population (highland cattle) inhabiting a novel (lowland) environment and their ancestral population (lowland cattle) (Figure 1d).

### 2.4 Categorization

Gene classification to these categories was performed using parametric bootstrapping, following the methodology outlined in Wood et al. (2023). We conducted the categorization exclusively on genes that exhibited significant plastic change (PC) and/or evolutionary change (EC). In particular, genes were categorized into three groups based on their evolutionary response to ancestral plasticity: (1) Reinforcement: if EC × PC > 0, indicating that the initial plastic change has been amplified during subsequent evolutionary changes. (2) Overshooting: if EC × PC < 0 and |EC| < 0.5 × |PC|, suggesting that the plastic change was close to the optimal value for a specific trait. (3) Reversion: if EC × PC < 0 and |EC| > 0.5 × |PC|, signifying a reversal in the plastic change where the optimal value for a trait in a new habitat approaches its original value.

### 2.5 Functional annotation

We performed GO term enrichment analysis to identify enriched biological processes associated with the set of reversion genes by using the DAVID functional annotation tool. We considered GO categories that showed significant enrichment, with a false discovery rate (FDR) <0.05 and Bonferroni corrected *p* value < .05.

## 3 RESULTS

### 3.1 Genetic and plastic phenotypic changes between lowland and Tibetan cattle

**FIGURE 2:**
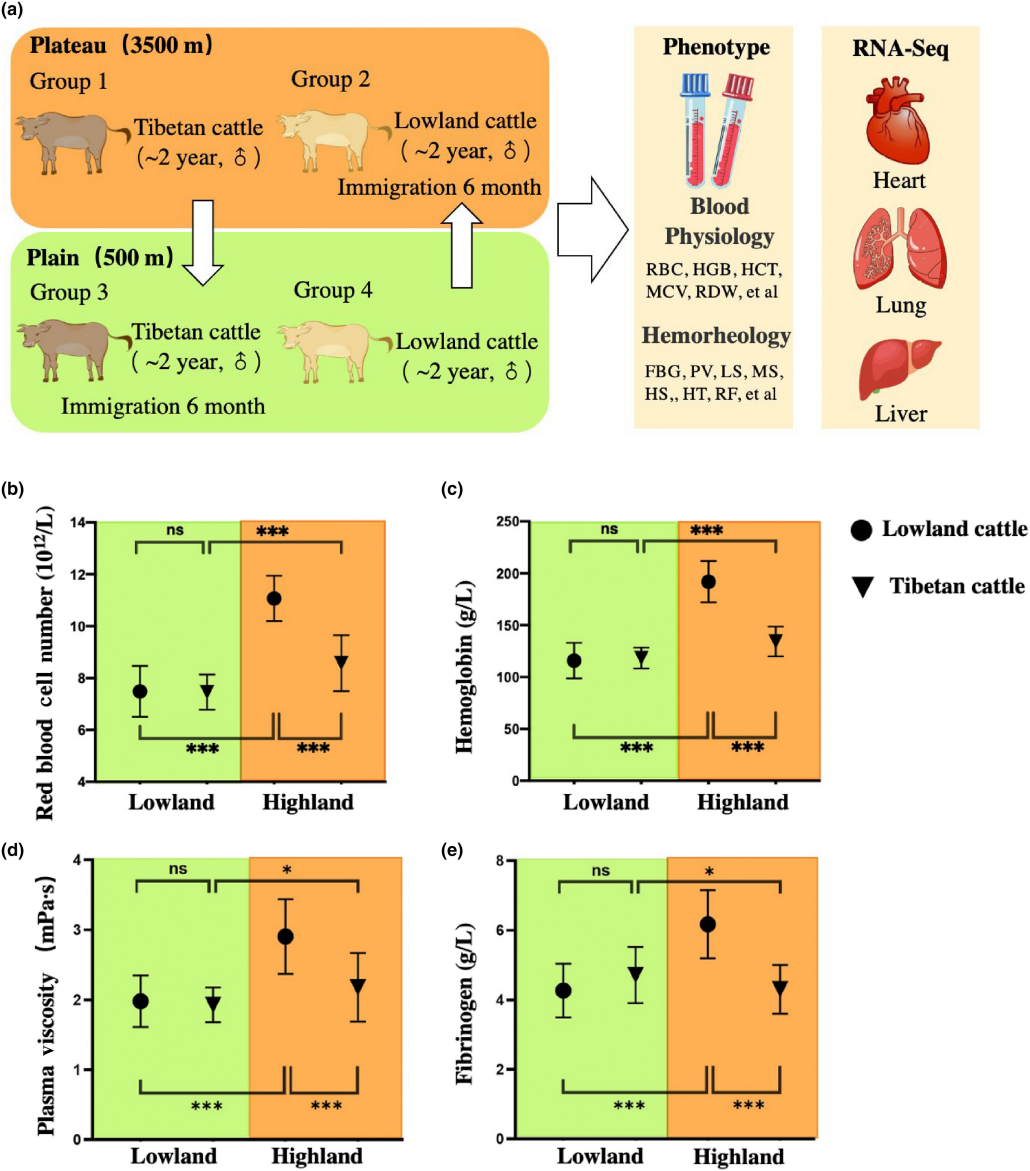
Reciprocal transplant experiment analysis the plastic and genetic changes between Tibetan and lowland cattle. (a) The experimental design involves conducting reciprocal transplant experiments, two cattle breeds were phenotyped and RNA‐sequenced in its native environment as well as the native environment of the other breed. Red blood cell number (b) and hemoglobin (c) are presented as indicators of blood physiology, plasma viscosity (d), and fibrinogen (e) are presented as indicators of blood hemorheology. Breeds are indicated by different symbols, while environments are indicated by different colors. Error bars show one standard error based on the binomial distribution. *p* values are determined by a *G* test of independence. ns, P > 0.06; *, 0.01 < P < 0.05; ***, P < 0.001.

**FIGURE 3:**
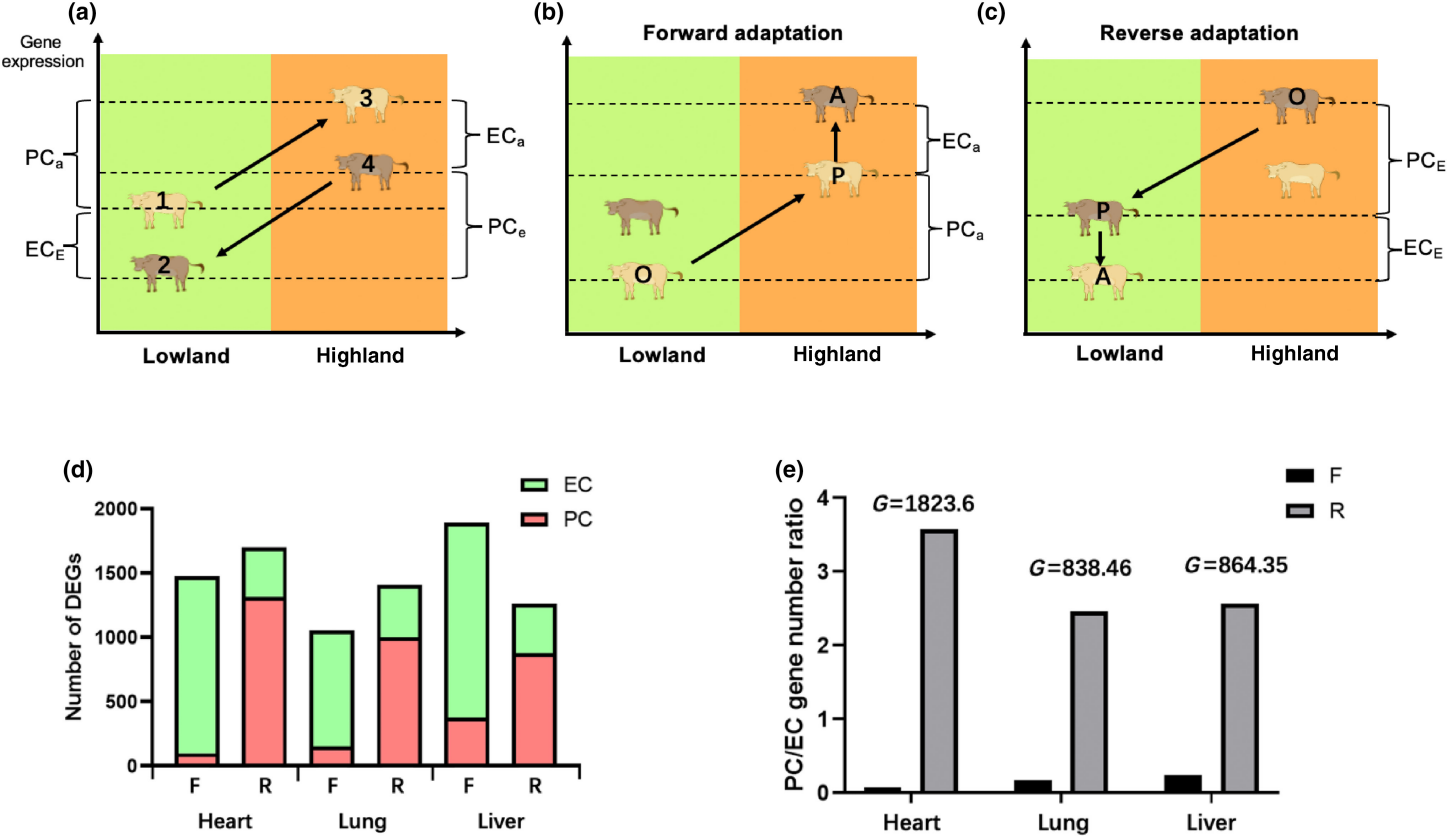
Reciprocal transplant experiments reveal plastic and genetic differences in gene expression levels between Tibetan and lowland cattle. (a) Samples 1 represent the lowland cattle in original lowland environment; samples 3 represent the lowland cattle in “foreign” highland environment; samples 4 represent the Tibetan cattle in “home” highland environment; samples 4 represent the Tibetan cattle in ancestral lowland environment. (b) Samples 1, 3, and 4, respectively, represent the original (O), plastic (P), and adapted (A) stages during the forward adaptation from the lowland to highland. (c) Samples 3, 1, and 2, respectively, represent the O, P, and A stages during the reverse adaptation from the highland to the lowland. (d) The numbers of differentially expressed genes (DEGs) in each tissue that undergo plastic changes (PC) and evolutionary changes (EC) during forward (F) or reverse (R) adaptation. (e) The ratio of the number of DEGs is PC divided by EC in F or R adaptation is calculated for each tissue and all tissues combined. *p* Values are determined using a *G* test of independence.

Reciprocal transplant experiments were used to analyze plastic and genetic phenotype changes in cattle altitude adaptation (Figure 2a). As Tibetan cattle were originally from lowland cattle and introduced to the Tibetan Plateau approximately 3600 years ago (Ajmone‐Marsan et al., 2010; Chen et al., 2015; Gao et al., 2017; Lei et al., 2006), we accordingly divided the stages of adaptation to analyze the phenotypic changes in different environments. We categorize the course of lowland cattle to the Tibetan Plateau as a form of forward adaptation, which we have labeled as groups 1, 2, and 3 in Figure 3a. These groups represent the initial stage of adaptation in the lowland (O, Figure 3b), followed by a stage of phenotypic plasticity in response to the highland environment (P, Figure 3b), and finally culminating in genetic evolutionary adaptations specific to the highland habitat (A, Figure 3b). The observed differences between stages O and P are primarily due to phenotypic plasticity, while disparities between stages P and A can be attributed to genetic evolution.

Conversely, when examining the reverse adaptation from Tibetan cattle back to their ancestral lowland environment depicted by groups 3, 4, and 1 in Figure 3c; we observe an original stage adapted for life in the highlands (O, Figure 3c), followed by a stage where phenotypic changes occur due to exposure to lowland conditions (P, Figure 3c), ultimately leading to full adaptation for survival in the lowlands (A, Figure 3c). We conducted an analysis of blood physiology and hemorheology, which are crucial to fitness, to analyze phenotypic changes in four experimental groups of cattle. The results show that a significant elevation in red blood cell (RBC) and hemoglobin (HGB) when lowland cattle were brought to highland suggests the hypoxia‐induced ancestral phenotype plasticity shifts the mean trait value (Figure 2b,c). However, a decreasing trend was observed in Tibetan cattle compared to lowland cattle when both are at high altitudes (Figure 2b,c), suggesting that the adaptation to highlands led to evolutionary genetic modifications and selective pressure based on genetic traits countered the plastic changes, ultimately restoring the ancestral phenotype. Additionally, in the lowland environment, Tibetan and lowland cattle show no significant difference in RBC and HGB (Figure 2b,c). The aforementioned observation, coupled with a substantial plastic change in the RBC and HGB of Tibetan cattle between lowland and highland environments (Figure 2b,c), suggests the presence of plastic change in blood biochemistry during potential reverse adaptation to lowland. Moreover, comparable patterns were also observed in other parameters of blood biochemistry and hemorheology, which are crucial for altitude adaptation (Figure 2d,e, Appendix S2).

### 3.2 Genetic and plastic changes in gene expression level between lowland and Tibetan cattle

We implemented a four‐stage procedure to assess the relative contributions of genetic and plastic changes in the expression differences. Initially, we assessed the expression levels of each differentially expressed gene (DEG) at stages O, P, and A as Lo, Lp, and La, respectively (Figure 3b). In the second stage, if there was a significant difference between Lo and Lp, it was categorized as ancestral plasticity change (PCa). In the third stage, if Lp and La exhibited a significant difference, we categorize it as ancestral evolution change (ECa). Finally, we applied a similar classification method to assign DEGs into evolved plasticity change (PCe) and evolved evolution change (ECe) for the reverse adaptation to the lowland (Figure 3c).

The results of our study indicate that the alterations in altitude environment have a significant impact on both genetic and plastic changes in gene expression (Table 1). In the forward adaptation course, the plastic changes of lowland cattle (PCa) exhibited the fewest number of DEGs, whereas a higher number of DEGs were observed in evolution change (ECa), suggesting that genetic changes play a predominant role in forward adaptation. However, reverse adaptations show a lower abundance of evolved evolutionary change (ECe) but a higher prevalence of evolved plasticity change (PCe) genes, which show an opposite trend compared to the forward adaptation (Figure 3d). By calculating the ratio of the number of PC genes to EC genes (PC/EC), we observed that the *R*_PC/EC_ in reverse adaptation is 47.9, 14.6, and 10.39 times higher than the ratio observed in the forward adaptation in heart, lung, and liver, respectively (*p* < 10^−16^, *G* test of independence; Figure 3e), indicating that plastic changes play a more prominent role in readapting to ancestral environments compared to adapting to novel environments. The frequency of plastic expression alterations is comparatively more pronounced in reverse adaptations than in forward adaptations, suggesting that the reaction norm of highland‐adapted cattle deviates from that of lowland‐adapted cattle, resulting in a non‐parallel shift in the slope of reaction norms (indicated by arrows) (Figure 1d).

**TABLE 1:** Significantly differently expressed genes.

	Total	Up	Down
*Ancestral plasticity change (PCa)*			
Heart	118	133	55
Lung	221	192	29
Liver	639	410	229
*Ancestral evolutionary change (ECa)*			
Heart	1314	961	353
Lung	901	798	103
Liver	1518	1050	468
*Evolved evolutionary change (ECe)*			
Heart	386	292	94
Lung	407	331	76
Liver	342	257	85
*Evolved plasticity change (PCe)*			
Heart	1558	959	559
Lung	1149	970	179
Liver	997	834	163

*Note*: Different plasticity gives the number of genes with significantly different plastic responses in species from different environments. Ancestral plastic change gives the number of genes that changed expression between lowland cattle in lowland and highland environments. Ancestral evolutionary change gives the number of genes that changed expression between lowland cattle and Tibetan cattle in a highland environment. For evolved differences, Tibetan cattle and lowland cattle are compared within condition lowland environment, representing evolved evolutionary change. Evolved plasticity represents the number of expression‐changed genes in Tibetan cattle between lowland and highland environments.

### 3.3 Evolutionary responses generally reverse ancestral plasticity during the process of adaptation

**FIGURE 4:**
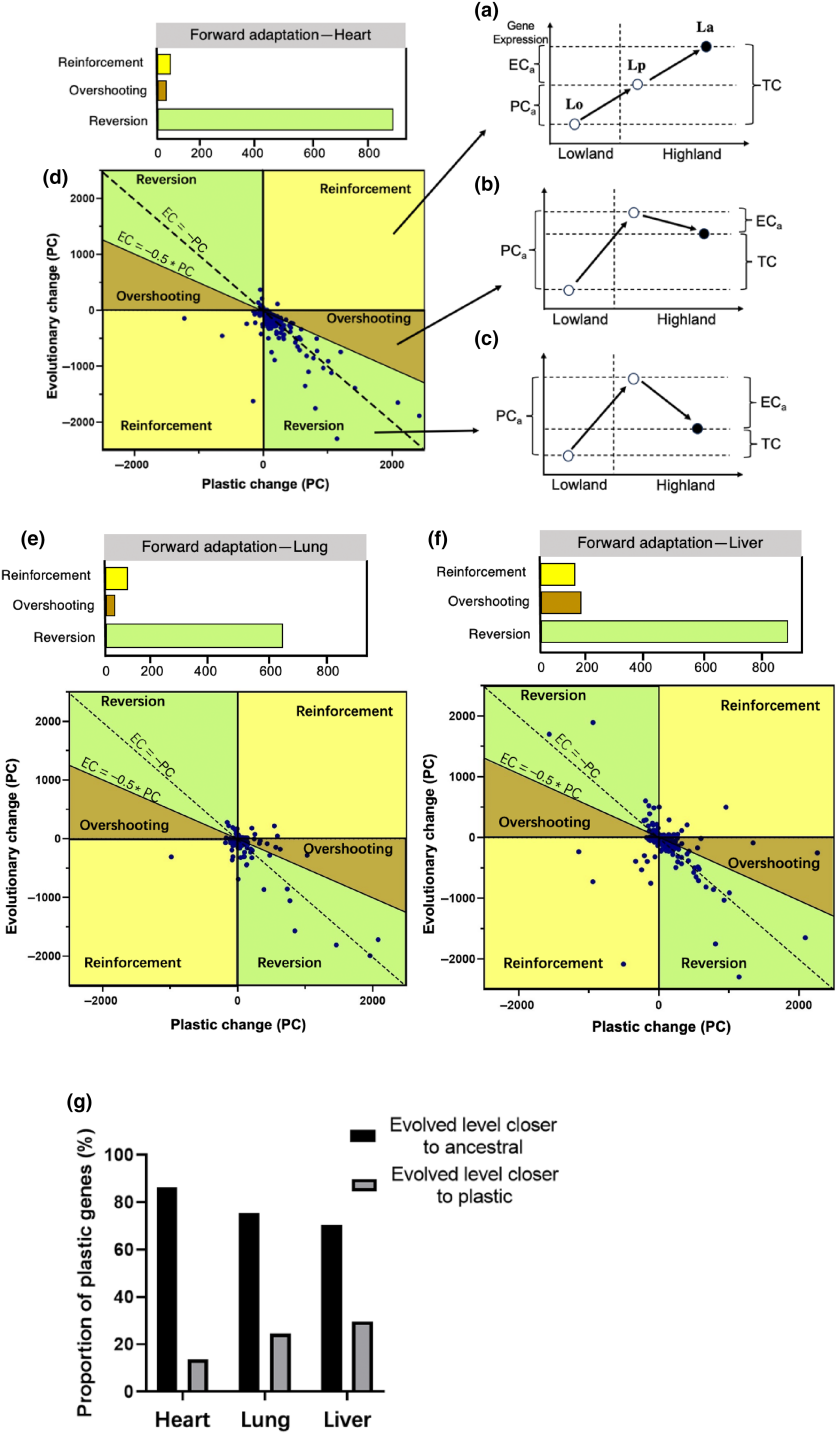
Impact of evolutionary responses to ancestral plasticity. (a–c) Cartoon representations of three categories of evolutionary response to ancestral plasticity. Dashed line represents transition from ancestral lowland environment to novel highland environment and associated trait shift. When an ancestral lowland population encounters to highland environment, an immediate phenotypic change occurs, shifting the trait from its initial value of Lo in the lowland environment to Lp in the highland environment. As populations undergo adaptation over time, a subsequent evolutionary change takes place, further shifting Lp to a new value of La. The evolutionary response to ancestral plasticity can be categorized into three distinct groups based on the values of PC and EC. (a) Reinforcement: The subsequent evolutionary change (EC) aligns with the direction of phenotypic change (PC). (b) Overshooting: PC has brought the trait value closer to the new optimum, where La is now closer to Lp than Lo. (c) Reversion: The optimal trait value in the new habitat is closer to the unstressed ancestor's value in its original environment (Lo), rather than its response (Lp). (d–f) The proportion of evolutionary responses to ancestral plasticity in heart, lung, and liver, respectively. Top bar plots showing numbers of genes displaying reversion, overshooting, and reinforcement. (g) Proportion of genes with expression levels after evolution (adapted stage La) are closer to ancestral levels (Lo), or closer to plastic levels (Lp).

To investigate the relationship between plasticity and adaptation, we conducted a comparative analysis of the initial plastic response exhibited by an ancestral population when exposed to a new environment (lowland cattle in lowland and highland conditions)—referred to as plastic change (PCa = Lp − Lo), and the subsequent genetic evolution in gene expression observed between the ancestral population and adapted population to new environment (lowland cattle and Tibetan cattle in highland conditions)—referred to as evolutionary change (ECa = La − Lp) (Figure 3b). The association between PC and EC can be categorized into three distinct patterns: (1) “reinforcement,” where both initial PC and subsequent EC drive expression toward the new optimum in the same direction (Figure 4a); (2) “overshooting,” where PC initially exceeds the new optimum, followed by EC adjusting expression in the opposite direction (Figure 4b); and finally, (3) “reversion,” where the new optimum aligns closer with ancestral levels of expression (Figure 4c). During reinforcement and overshooting scenarios, ancestral plasticity aids in fine‐tuning gene expression toward an optimal state for adapting to novel environments. Conversely, reversion events are likely to occur when ancestral plasticity becomes disadvantageous.

To examine whether ancestral plasticity promotes adaptive evolution, we assess the occurrence of reversion, reinforcement, and overshooting in the transcriptome‐wide patterns of three organs. To mitigate the risk of erroneous categorization due to minimal alterations in expression, only genes exhibiting substantial changes in PC and EC were placed into these three categories. The results demonstrate consistent patterns across the entire transcriptome in ancestral plasticity within three different tissues. In the heart, lung, and liver, the genes were classified as “reversion” and exhibited the majority proportion of genes exhibiting further evolutionary change in the direction of evolutionary plasticity, respectively (Figure 4d–f). Subsequently, we conducted a binomial test to evaluate whether the proportion of genes classified as either “reinforcement” or “reversion” significantly deviated from an expected equal distribution (50% for each category). Our extensive analysis of the transcriptome revealed a significant deviation from the expected 50% distribution for both categories. Specifically, there was a significantly higher percentage of genes exhibiting “reversion” (*p* < .001), while the percentage of genes demonstrating “reinforcement” was significantly lower than anticipated (*p* < .001). These findings suggest an uneven distribution of genes between these two categories, with reversion playing a dominant role in transcriptome plasticity. Consequently, ancestral plasticity is maladaptive for long‐term adaptation and fails to converge gene expression toward the new optimum in all three oxygen‐sensitive organs. This result aligns with previous research conducted on animals and plants, consistently demonstrating the prevalence of reversion (Ho & Zhang, 2018; Koch & Guillaume, 2020; Swaegers et al., 2020). For genes exhibiting ancestrally plasticity, approximately 86.36%, 75.35%, and 70.38% of genes show that the evolved level of the adapted population (La) was closer to their ancestral expression in the original environment (Lo) than to the plastic response level in highland environments (Lp) in heart, lung, and liver, respectively (Figure 4g). Therefore, the transcriptome analysis supports observed trends in phenotypic data by indicating that restoration of ancestral phenotypes is a prevailing tendency in gene expression evolution during novel environment adaptation in cattle.

### 3.4 Functional annotation of reversed plastic genes

For the entire transcriptome in the heart, enriched GO terms of the genes that underwent reversion were related to the respiratory chain and electron transport, transcription regulation, and ubiquitin conjugation (Figure S3). In the lung, the plastic genes that underwent reversion related to DNA damage and repair, steroidogenesis, and transcription regulation (Figure S4). In the liver, the plastic genes that underwent reversion related to the inflammatory response and immunity, cell adhesion, and lipid metabolism (Figure S5). Interestingly, the result of the functional annotation cluster of the genes that underwent reversion in heart and lung significant enrichment in protein post‐translational modification (such as Ubiquitin, SUMOylation, UFMylation), indicating the important role of post‐transcriptional regulation in shaping phenotypic plasticity.

## 4 DISCUSSION

In this study, we analyze the plastic and evolved responses in gene expression of cattle in response to the new environment by using a reciprocal transplant approach of cattle adapted to the Tibetan Plateau and adjacent lowlands. We reported a tendency for cattle to restore ancestral patterns during new environment adaptation. At the transcriptomic level, a nonparallel change in the slope of reaction norms was observed between Tibetan and lowland cattle. The ancestral plasticity is more frequently reversed than reinforced during adaptation to a highland environment. A high proportion of the originally plastic genes evolved to expression levels that were closer to original levels than to ancestrally plastic levels. The pattern in phenotypic blood fitness traits was congruent with expression data, as more initial plastic change traits were also reversed, supporting the view that ancestral plasticity is maladaptive for long‐term adaptation.

The reaction norm of Tibetan cattle differs from that of lowland cattle, potentially as a consequence of evolutionary adaptation to high‐altitude environments. During the process of forward adaptation to the highland environment, numerous genetic transcriptomic changes were observed. However, when Tibetan cattle were reintroduced to the lowland ancestral environment, it became apparent that plastic changes played a predominant role in reshaping their transcriptomes, effectively bringing them back to a state that was more suitable for the lowland conditions. The trend identified in our study along with highly similar patterns in comparable experiments of *Escherichia coli*, guppies, and chickens (Ho et al., 2020). Even in cattle, more advanced mammals with longer domestication, the return to ancestral lowland environments results in a higher R_PC/EC_ compared to adaptations to new environments, suggesting a broad applicability of this trend among species. Although this observation does not imply that no genetic adaptation is required upon organisms' return to their ancestral environment, it does suggest that fewer genetic phenotypic changes are necessary for readaptations to ancestral environments compared to adaptations in novel environments. This asymmetry can be understood as the fact that to adapt to the plateau environment, Tibetan cattle evolved a large number of genetic changes, including a proportion of transcriptional regulation factors that only function in highland environment, but not ancestral lowland environments, such as hypoxic response regulatory elements (HREs) and its downstream regulated target genes. Hence, causes changes in the slope of reaction norms of Tibetan cattle different from that of lowland cattle.

Different patterns have been documented that describe the relationship between plasticity and evolved changes in gene expression. The perspective that phenotypic plasticity is advantageous can be categorized into two groups: (1) it aids organismal survival in new environments, serving as a stepping‐stone toward genetic adaptation, as adaptive evolution cannot occur if individuals are unable to survive under stress from new environmental conditions (Kenkel & Matz, 2016; Mäkinen et al., 2016; Mallard et al., 2020). (2) Phenotypic plasticity brings phenotypes closer to the new optimum by aligning both plastic and evolutionary responses in the same direction (Li et al., 2017). However, some other research suggests that plasticity may be maladaptive. The subset of genes that exhibit both adaptive differences and phenotypic plasticity show counter gradient variation in expression levels in *Fundulus heteroclitus* adapting to different temperatures, in *Drosophila* adapting to different diets (Huang & Agrawal, 2016; Yampolsky et al., 2012) and in guppies adapting to different predation environments (Ghalambor et al., 2015). The findings from multiple experimental evolution studies and computational metabolic network analysis demonstrate that genetic changes more frequently reverse rather than reinforce plastic phenotypic changes in nearly all adaptations (Ho & Zhang, 2018). Our study supports these previous observations, as we have discovered a large proportion of reversions rather than reinforcements in all three oxygen‐sensitive organs, suggesting that plasticity was mostly maladaptive in long‐term adaptation. In addition, we considered the possibility of plastic changes not only reverting to their original state but also moving expression levels closer to the adapted level (La) rather than the ancestral level of expression in the lowland (Lo). Conversely, suppose we observe that the adapted level of expression (La) is closer to the original level (Lo, lowland cattle in a lowland environment). In that case, it indicates maladaptive plastic responses as they moved expression levels further away from the new optimum and were compensated for during evolution (refer to Figure 1c). Our findings indicate that Tibetan cattle that have adapted exhibit a significant proportion of expression levels closer to their ancestral level (Lo) compared to their ancestral plastic response (Lp).

At the phenotypic level, our results supported the reverse role of plasticity in adaptation to highland environment. Indeed, we found that most of the biochemical and hemorheology traits tend to restore ancestral patterns in Tibetan cattle. The red blood cells serve as an example, where an increase in RBC levels is a common physiological response observed in lowland species during short‐term exposure to high‐altitude environments. However, high concentrations of red blood cells are harmful to the body and are not suitable for long‐term adaptation. The mechanism may be that the plasticity phenotypic changes may confer benefits in response to short‐term exposure to a new environment, as plastic phenotypic changes represent emergency stress responses that enhance survival for organisms. However, after long‐term exposure, emergency stress responses driven by plasticity may become maladaptive and reversed by genetic compensation (Grether, 2005). One well‐documented instance is hypoxic pulmonary hypertension (HPH), an adverse response to chronic oxygen deprivation experienced by lowland individuals living at high altitudes, yet it shows mitigation in indigenous populations residing in such regions (Monge et al., 1992). At sea level, individuals experience constriction of their pulmonary arterial vessels when exposed to low levels of oxygen, which promotes regional variation in lung ventilation and blood flow, thereby enhancing the efficiency of gas exchange. However, this vasoconstriction becomes counterproductive at higher altitudes due to widespread lung oxygen deprivation. Consequently, there is a remodeling and thickening of pulmonary vessels that reduces their flexibility and increases the pressure within the pulmonary arteries (Shimoda & Laurie, 2014; Sylvester et al., 2012). HPH can impede the uptake of oxygen and, in severe cases, lead to life‐threatening conditions such as pulmonary edema, hypertrophy of the right ventricle, and heart failure (Sylvester et al., 2012). However, these pathological responses to chronic oxygen deprivation are absent among Tibetan and several other species inhabiting high‐altitude regions, similar pressures were observed within their pulmonary arteries as those living at sea level. Previous studies on high‐altitude physiological adaptation (HPH) have mainly focused on investigating high‐altitude species in their natural environment, making it difficult to determine whether there has been evolved a loss of plasticity or the preservation of plasticity with a downward shift in trait average. A similar trend was observed in deer mice in controlled environments providing evidence that supports both possibilities (Velotta et al., 2018). When exposed to chronic hypoxia, lowland native deer mice show significant increases in pulmonary arterial pressure, leading to hypertrophy of the right ventricle. On the other hand, these responses are less pronounced among highland native deer mice due to a shallower range of reactions for both traits combined and specifically for pulmonary arterial pressure. This provides a physiological demonstration of how individuals from high‐altitude regions appear to have evolved mechanisms that mitigate unfavorable plastic responses to chronic hypoxia. Our physiological studies of lowland and Tibetan cattle provide potential examples of genetic assimilation of adaptive plasticity as well as genetic compensation of maladaptive plasticity.

Our finding contributes to the ongoing debate on the relative roles of plasticity and genetic changes in environment adaptation and demonstrates that evolutionary adaptations generally reverse phenotypic plasticity to restore ancestral phenotypes during cattle new environment adaptation.

## AUTHOR CONTRIBUTIONS

**Qiang Jiang:** Investigation (equal); writing – original draft (equal). **Li Zhu:** Data curation (equal); resources (equal). **Hao Zeng:** Formal analysis (equal); resources (equal). **Zhuzha Basang:** Investigation (equal); resources (equal). **Quji Suolang:** Data curation (equal); resources (equal). **Jinming Huang:** Funding acquisition (lead); supervision (lead). **Yafei Cai:** Funding acquisition (lead); supervision (lead).

## CONFLICT OF INTEREST STATEMENT

The authors declare that the research was conducted in the absence of any commercial or financial relationships that could be construed as a potential conflict of interest.

## Supporting information


Appendix S1.
Appendix S2.
Appendix S3.
Appendix S4.
Appendix S5.

## Data Availability

TPM values of the cattle RNA‐seq data generated and analyzed were provided in Appendix S1; Biochemical and hemorheology values were provided in Appendix S2.
