# Supplementary material for: Evolutionary adaptations generally reverse phenotypic plasticity to restore ancestral phenotypes during new environment adaptation in cattle
Source: Ecol Evol. 2024 Jun 4;14(6):e11489. doi: 10.1002/ece3.11489 (PMC11150418; doi:10.1002/ece3.11489)
Supplement: Supplementary file 1 — Appendix S1. Appendix S2. Appendix S3. Appendix S4. Appendix S5. [file ECE3-14-e11489-s001.zip › Supplement S2-S5 Caption Text.docx]

Supplement S2-S5 Caption Text

Fig S3：Gene classification and functional enrichment in heart

Fig S4：Gene classification and functional enrichment in lung

Fig S5：Gene classification and functional enrichment in liver
